# Supplementary material for: Content-rich biological network constructed by mining PubMed abstracts
Source: BMC Bioinformatics. 2004 Oct 8;5:147. doi: 10.1186/1471-2105-5-147 (PMC528731; doi:10.1186/1471-2105-5-147)
Supplement: Additional File 5 — The original Chilibot query results of the term "long-term potentiation (LTP)" and 22 other terms, limiting the latest references analyzed to the years 1990, 1995, 2000, and 2004. [file 1471-2105-5-147-S5.bz2 › chilibotAdditionalFile5/ltp2004/html/TRKB.html]

 


**TRKB** (Input: TRKB ) 

---


**Google Searches:** Entire Web  | EDU domain only  | PDF files only

.

**External Links:** OMIM |
LocusLink
| Swissprot
| GeneCards

  
**Maps of TRKB**

Simple
Complete
graph in
radiant
tree
square
layout.

**New Hypothesis !**

**Synonyms** 

- trk b   [PubMed]

- trkb   [PubMed]

**Synopsis**

- Glutamate release was enhanced, and the data suggest that this may be a consequence of increased activation of **TrkB** and ERK.  Behav Neurosci, 2002 
  [22]

- Activation of **trkB** modulates hippocampal long term potentiation  [LTP] , in which postsynaptic N methyl D aspartate glutamate receptors play a key role.  Proc Natl Acad Sci U S A, 1997 
  [19]

- Our findings suggested that activation of **TrkB** PI3 K and protein synthesis signaling pathway by BDNF in the hippocampus is important for spatial memory.Molecular Psychiatry 17 224.  Mol Psychiatry, 2003 
  [19]

- These findings suggest that BDNF **TrkB** signaling in the hippocampus plays a crucial role in learning and memory.  J Pharmacol Sci, 2003 
  [19]

- It is concluded that BDNF **TrkB** signaling plays an important role in learning and memory.  Life Sci, 2002 
  [19]

- showing brain derived neurotropic factor modulates LTP by binding to **TrkB** receptors on the postsynaptic neuron.  Science, 2002 
  [19]

- **TrkB** IgG or K252a itself completely blocked LTP, suggesting that endogenous BDNF or another **TrkB** ligand plays a role in LTP in the developing visual cortex.  J Neurosci, 1997 
  [19]

- Our findings suggest the importance of interaction between BDNF **TrkB** signaling and NMDA receptors for spatial memory in the hippocampus.  Learn Mem< MedlineTA>Learn Mem, 1997 
  [19]

- A recent study using mice carrying a mutation in a specific tyrosine residue of **trkB** pinpoints a necessary role for the phospholipase Cgamma pathway in CREB activation and LTP maintenance.  Trends Neurosci, 2003 
  [19]

- Firstly, we show that while BDNF and **trkB** are induced as immediate early genes following injury, the injury induced expression of activinA and trkC may be regulated by ITFs.  Prog Neurobiol, 1999 
  [16]

- Taken together, these results indicate that endogenous Trk B  [**TRKB**]  ligands are necessary for the expression of LTP  Eur J Neurosci, 2000 
  [14]

- Our results provide genetic evidence that **TrkB** mediates hippocampal plasticity via recruitment of PLCgamma, and by subsequent phosphorylation of CaMKIV and CREB.  Neuron, 2002 
  [14]

- However, both **TrkB** IgG fusion proteins and BDNF antibody similarly reduced LTP, suggesting that only BDNF but no other ligands of the **TrkB** receptor are likely to be involved in LTP induction.  J Neurosci, 1999 
  [13]

- We found that homo and heterozygous mutant mice show no difference in the induction rate or magnitude of E LTP and L LTP induced by theta burst or tetanus stimulation, suggesting that the Shc binding site in the **TrkB** receptor and its downstream activated signaling cascade is NOT involved in hippocampal synaptic plasticity.  Neuropharmacology, 2000 
  [13]

- In fact, BDNF potentiates intracellular calcium release presynaptically and BDNF induced **TrkB** activation results in phosphorylation of synapsin I via mitogen activated protein kinase, which increases the number of synaptic vesicles available for release.  Sheng Li Xue Bao, 2003 
  [11]
